# Association of vaginal microbiome, cytokines, and spontaneous preterm birth among Chinese women: a nested case-control study

**DOI:** 10.1128/spectrum.02607-25

**Published:** 2026-02-27

**Authors:** Yefang Ke, Ying Sun, Jun Wu, Lina Ye, Zhe Zhu

**Affiliations:** 1 Department of Clinical Laboratory, Women and Children's Hospital of Ningbo University 36982, Ningbo, Zhejiang, China; 2 Ningbo Key Laboratory of Prevention and Treatment of Embryonal Diseases, Ningbo, Zhejiang, China; 3 Department of Obstetrics, Women and Children's Hospital of Ningbo University 36982, Ningbo, Zhejiang, China; 4 Department of Gynecology, Women and Children's Hospital of Ningbo University 36982, Ningbo, Zhejiang, China; 5 Department of Blood Transfusion, Ningbo No.2 Hospital 74782, Ningbo, Zhejiang, China; Weill Cornell Medicine, New York, New York, USA

**Keywords:** spontaneous preterm birth, vaginal microbiome, cytokines, *Lactobacillus iners*, IL-6

## Abstract

An imbalance of the vaginal microbiome and dysregulation of cytokines are associated with spontaneous preterm birth (sPTB). To date, the relationship between the vaginal microbiome, cytokines, and sPTB remains unclear in the Chinese population. Herein, we conducted a nested case-control study using data from a prospective cohort of 749 Chinese women with a singleton pregnancy who were enrolled between 16 and 28 weeks of pregnancy. Cases consisted of individuals experiencing sPTB (*n* = 38), while controls were selected randomly at a 4:1 ratio to cases (*n* = 152). Compared to the term group, the sPTB group exhibited significantly increased abundance of vaginal *Aerococcus christensenii*, *Gardnerella swidsinskii*, and *Lactobacillus iners*, along with elevated levels of interleukin (IL)-1β, IL-6, and IL-12p70 in vaginal fluid (*P* < 0.05). Least absolute shrinkage and selection operator (LASSO) regression identified *L. iners*, *G. swidsinskii*, and IL-6 as significant risk factors for sPTB, with adjusted odds ratios (ORs) (95% CI) of 1.57 (1.06–2.34), 1.45 (1.03–2.05), and 2.05 (1.43–2.93), respectively. Finally, a logistic regression model for sPTB was established incorporating *L. iners*, *G. swidsinskii*, and IL-6, which yielded an area under the receiver operating characteristic curve (AUC) of 0.73. These findings suggest that alterations in the vaginal microbiome and cytokine levels may contribute to sPTB in the Chinese population.

**IMPORTANCE:**

Preterm birth (PTB) is the leading cause of death in children under 5 years of age, of which about 70% were spontaneous ones (sPTB); while genitourinary infections are implicated in 25–40% of sPTB cases. Previous studies have revealed some features of vaginal microbiome and cytokines related to sPTB: increased richness and diversity, increased levels of *Lactobacillus iners*, BV-associated bacteria, low abundance of *L. crispatus*, and high levels of pro-inflammatory cytokines. However, there were also some inconsistent findings, and little is known in the Chinese population. This study confirmed the correlations between vaginal microbiome, cytokines, and sPTB in Chinese pregnant women. Specifically, elevated vaginal *L. iners, G. swidsinskii*, and IL-6 levels were significantly associated factors, which may help to identify women at high risk of sPTB.

## INTRODUCTION

Preterm birth (PTB) is the leading cause of death in children under 5 years of age, with a rate of 11% globally in 2019 (1). Spontaneous preterm birth (sPTB) accounts for 70% of all PTB cases (2); while genitourinary infection is considered a significant cause, implicated in 25–40% of sPTB cases (3).

Previous studies have identified vaginal microbiome signatures associated with sPTB, including increased richness and diversity (4), depletion of *Lactobacillus* species (particularly *Lactobacillus crispatus*) (5), and dominance of Community State Type (CST) III (*Lactobacillus iners-*dominated) (6) or CST IV (highly diverse group) (7). Nevertheless, these associations were not always consistent across studies. Findings often varied even among different racial groups within the same study, as the vaginal microbiome composition differs among women of different ancestries (8, 9). Compared to women of European ancestry, who exhibit a higher abundance of *L. crispatus*, women of African ancestry show increased vaginal microbiome diversity, along with higher levels of *L. iners* and non-*Lactobacillus* species, and experience a higher rate of preterm birth (8).

Beyond microbial composition, vaginal dysbiosis can trigger inflammatory responses, resulting in a hyper-inflammatory state characterized by elevated levels of inflammatory cytokines in women experiencing sPTB (5, 10–12).

Numerous studies on the vaginal microbiome and preterm birth have focused on women of European and African ancestry (5, 7, 8, 10, 13–16); it is still largely unknown in the Chinese population. In China, the preterm rate is lower than that reported globally (17), but the absolute number of preterm births was the second largest all over the world (18). Moreover, the preterm rate in China has shown an upward trend: as reported by the Lancet Global Health, it increased from 5.9% in 2012 to 6.4% in 2018 (17); similarly, according to another nationwide survey, it rose from 5.13% in 2017 to 6.56% in 2022 (19). Therefore, we conducted a nested case-control study to explore the relationship between vaginal microbiome and sPTB in Chinese pregnant women. Additionally, cytokines with pro-inflammatory properties that contribute to sPTB pathogenesis were also investigated.

## RESULTS

### Characteristics of the study population

Demographics and characteristics of study subjects are shown in Table 1. All participants were of Han Chinese nationality, with a mean age of around 30 years old. There were no significant differences in the race, educational level, parity, mode of conception, gestational age (GA) at enrollment, mode of delivery, and fetal sex between groups. Although mean age did not differ significantly, the sPTB group had a higher proportion of women >35 years (38.9% vs 10.5%, *P* = 0.008). Additionally, the sPTB group had a higher previous miscarriage rate, though the difference was not significant (55.3% vs 37.5%, *P* = 0.071). As expected, GA at delivery and fetal birth weight were significantly lower in the sPTB group (*P* < 0.001). Among 38 cases with sPTB, 17 cases delivered <28 weeks of GA (extremely sPTBs).

**TABLE 1:** Demographics and characteristics of study population^*a*^

	Term (*n* = 152)	sPTB (*n* = 38)	*P* value
Race			
Chinese	152 (100)	38 (100)	–^*d*^
Age			
Mean (SD)	29.8 (4.0)	30.9 (4.7)	0.14
>35 years	16 (10.5)	11 (28.9)	0.008
Previous miscarriage	57 (37.5)	21 (55.3)	0.071
Education level			
Primary/junior high school	39 (25.7)	10 (26.3)	0.934
High school	17 (11.2)	6 (15.8)	0.436
College or above	96 (63.2)	22 (57.9)	0.55
Parity			
0	103 (67.7)	24 (63.1)	0.59
1	39 (25.6)	10 (26.3)	0.934
≥2	10 (6.6)	4 (10.5)	0.627
Mode of conception			
Naturally conceived	136 (89.5)	34 (89.5)	1
Assisted reproduction	16 (10.5)	4 (10.5)	1
Gestational age in weeks (IQR)			
At enrollment	22.0 (18.0–24.0)	21.0 (20–22.0)	0.204
At delivery	39.0 (38.0–39.0)	28.0 (23.0–34.3)	<0.001
Mode of delivery			
Vaginal delivery	94 (61.8)	29 (76.3)	0.095
Cesarian section	58 (38.2)	9 (23.7)	0.095
Fetal birth weight (g, IQR)^*b*^	3,260 (3,050–3,550)	1,500 (640–2,300)	<0.001
Fetal sex^*c*^			
Female	77 (50.6)	16 (42.1)	0.821
Male	75 (49.3)	17 (44.7)	0.821

^*a*^ Continuous data were compared using the Student's *t* test (normal distribution) or the Mann-Whitney *U* test (non-normal distribution). Categorical data were analyzed by the chi-square or Fisher's exact test. *P* values < 0.05 were considered significant. sPTB: spontaneous preterm birth.

^*b*^ Fetal birth weight of three preterm infants was not available.

^*c*^ The fetal sex of five preterm infants was not available.

^*d*^ "–", not applicable.

### Diversity and vaginal community state type comparison between the sPTB and term groups

**Fig 1:**
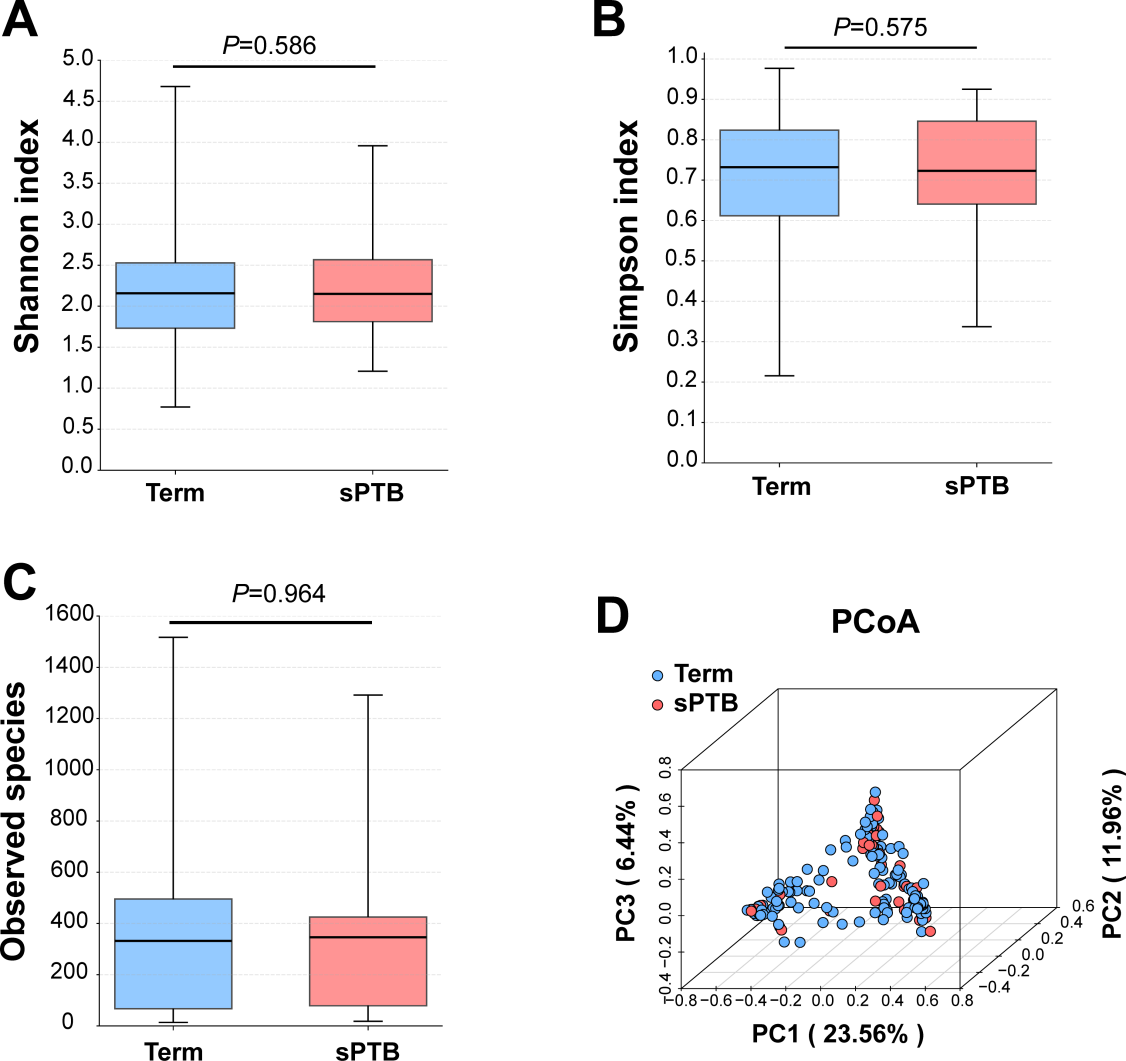
Species diversity between the term and sPTB groups. Alpha diversity comparison for (**A**) Shannon index, (**B**) Simpson index, and (**C**) Observed species. The bounds of the box represent the first and third quartiles, the center line represents the median, and whiskers show min-to-max values. Significant differences were calculated using the Mann-Whitney *U* test, *P* values < 0.05 were considered significant. (**D**) PCoA of beta diversity based on Bray-Curtis dissimilarity. Samples are colored by gestational age group: Term (blue) and sPTB (red). CST, community state type; PCoA, principal coordinate analysis; sPTB, spontaneous preterm birth.

Alpha diversity indices, including Shannon and Simpson indices (representing diversity) and Observed species (representing richness), demonstrated no statistically significant differences between the term and sPTB groups (Fig. 1A through C). Specifically, the Shannon index was 2.16 (1.73–2.53) in the term group compared to 2.15 (1.81–2.57) in the sPTB group (*P* = 0.586). Similarly, the Simpson index showed values of 0.73 (0.61–0.82) versus 0.72 (0.64–0.85) in the term and sPTB groups, respectively (*P* = 0.575). For observed species, both groups demonstrated similar distributions (term: 427.75 [67.25–495.00] vs sPTB: 346.50 [79.00–425.50], *P* = 0.946). Meanwhile, PCoA based on Bray-Curtis distances showed no distinct clustering separating the term and sPTB groups (Fig. 1D).

**Fig 2:**
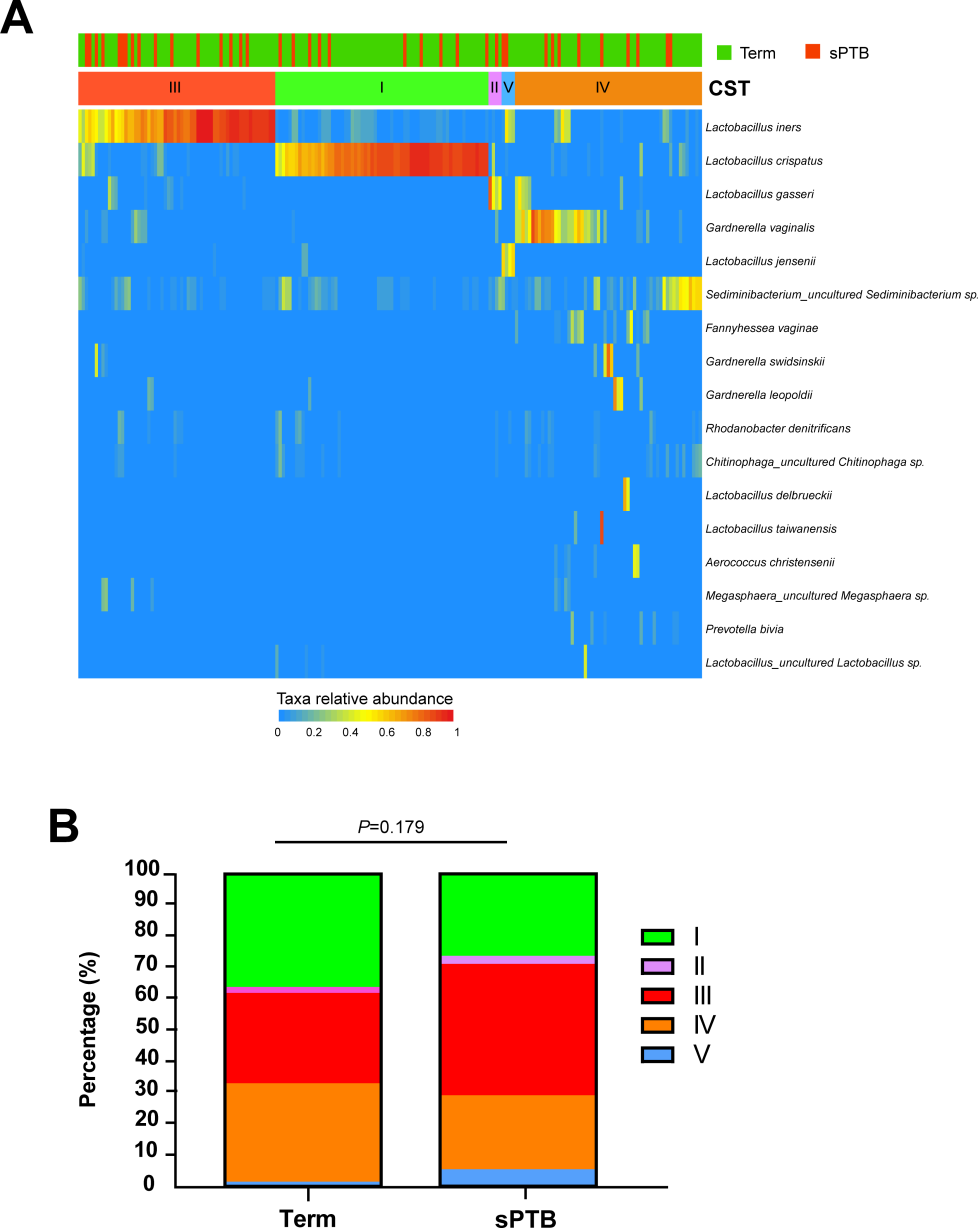
CST distributions in the term and sPTB groups. (**A**) Heatmap of microbial taxa relative abundance correlated with GA at delivery and CST. The bacterial taxa with the top 17 relative abundances in the vaginal microbiome are shown on the heatmap. (**B**) Comparison of CST composition between the term and sPTB groups. Stacked bar plots showing proportion of CSTs. Statistical significance based on Fisher’s exact test, *P* values < 0.05 were considered significant. CST, community state type; GA, gestational age; sPTB, spontaneous preterm birth.

Among all participants, overall microbiota profiles were predominantly composed of CST I, CST III, and CST IV—CST I: 34.2% (65/190), CST III: 31.6% (60/190), and CST IV: 30.0% (57/190). CST II and CST V represented only 2.1% (4/190) and 2.1% (4/190), respectively (Fig. 2A; Table S5).

In the term group, the distribution of CST was as follows: CST I (36.2%, 55/152), CST IV (31.6%, 48/152), CST III (28.9%, 44/152), CST II (2.0%, 3/152), and CST V (1.3%, 2/152). The distribution of CST in the sPTB group showed no statistically significant difference compared to the term group (*P* = 0.179). In the sPTB group, CST III was most prevalent (42.1%, 16/38), followed by CST I (26.3%, 10/38), CST IV (23.7%, 9/38), CST V (5.3%, 2/38), and CST II (2.6%, 1/38) (Fig. 2B).

### Comparison of bacterial taxa and cytokines between the sPTB and term groups

**Fig 3:**
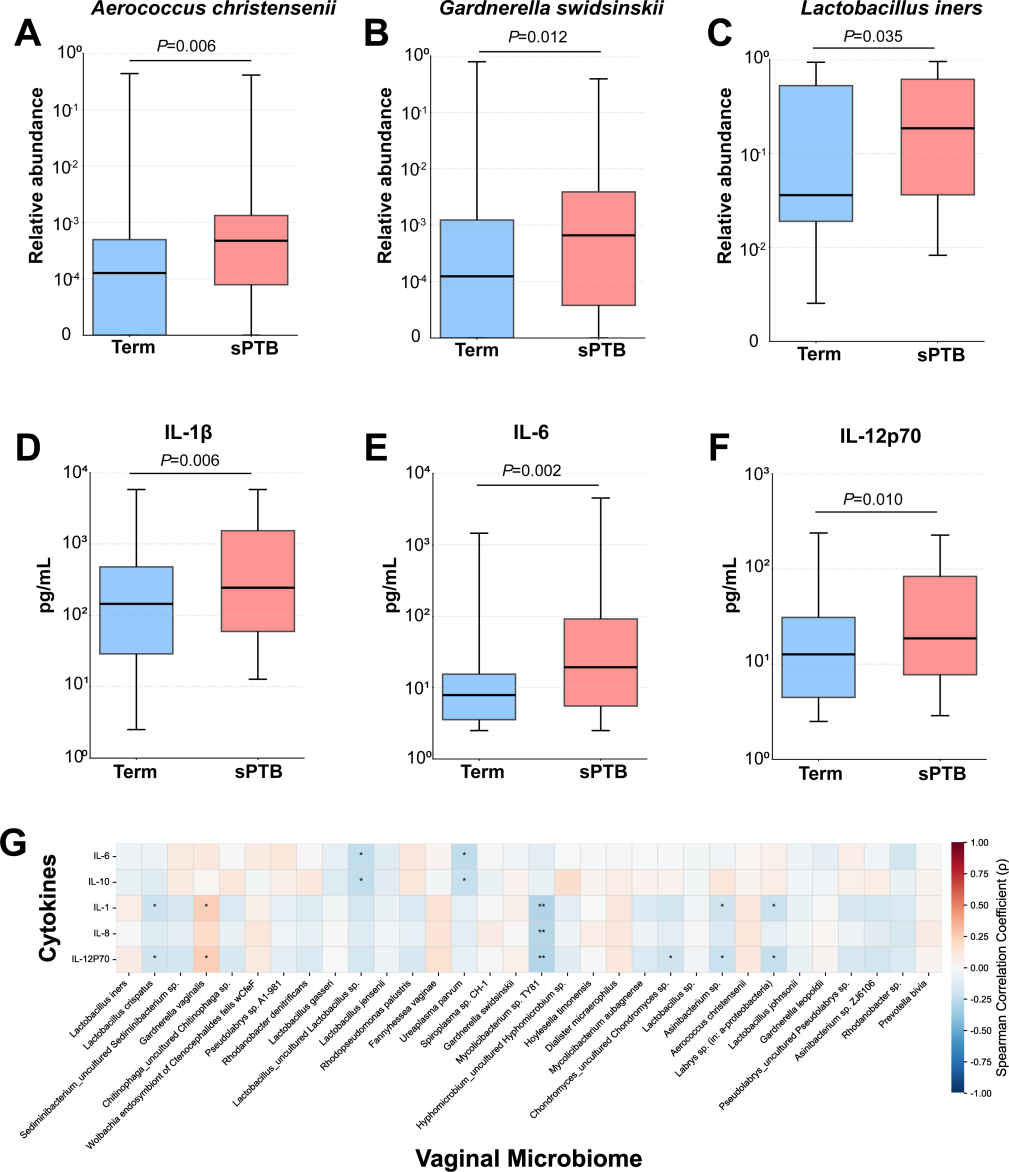
Vaginal microbiome and cytokine profiles in term versus sPTB: comparative analysis and correlations. Comparison of bacterial taxa and cytokines with statistically significant differences between the sPTB and term group (**A**) *Aerococcus christensenii*, (**B**) *Gardnerella swidsinskii*, (**C**) *Lactobacillus iners*, (**D**) IL-1β, (**E**) IL-6, and (**F**) IL-12p70. The bounds of the box represent the first and third quartiles, the center line represents the median, and whiskers show min-to-max values. Significant differences were calculated using the Mann-Whitney *U* test. *P* values < 0.05 were considered significant. (**G**) Correlations between bacterial taxa and cytokines in all study populations. Correlations were assessed using Spearman’s correlation, and FDR-adjusted *P* values < 0.05 were considered significant (*, *P* < 0.05, **, *P* < 0.01). sPTB, spontaneous preterm birth; IL, interleukin.

A total of 32 bacterial taxa met the inclusion criteria: a relative abundance of ≥1% in at least 5% of samples, or ≥0.1% in at least 15% of samples. The relative abundances of *Aerococcus christensenii*, *Gardnerella swidsinskii*, and *L. iners* were significantly lower in the term group compared with the sPTB group: (0.01 [0.00–0.05] % vs 0.05 [0.01–0.11] %, *P* = 0.006), (0.01 [0.00–0.12] % vs 0.07 [0.00–0.39] %, *P* = 0.012), and (3.61 [1.96–52.93] % vs 18.58 [3.65–61.71] %, *P* = 0.035), respectively (Fig. 3A through C). *G. swidsinskii* and *A. christensenii* were BV-associated bacteria that showed statistically significant differences between the two groups, and *L. iners* was the only *Lactobacillus* spp. that showed a statistically significant difference (Tables S6 and S7).

For IL-1β, IL-6, IL-8, IL-10, and IL-12p70, samples below the detection limit constituted less than 30% of the total samples, and these cytokines were selected for analysis. The term group had significantly lower levels of IL-1β (144.84 [28.74–495.83] vs 243.51 [49.82–1574.63] pg/mL, *P* = 0.006), IL-6 (7.86 (3.54–15.47] vs 19.29 [5.30–137.42] pg/mL, *P* = 0.002), and IL-12p70 (12.70 [4.44–31.14] vs 18.65 [7.08–86.45] pg/mL, *P* = 0.010) compared with the sPTB group (Fig. 3D through F; Table S8).

In all study populations, there were 17 significant associations between cytokines and bacterial taxa (Fig. 3G; Table S9). Notably, *G. vaginalis* was positively correlated with IL-1β and IL-12p70, while *L. cr*ispatus was negatively correlated with IL-1β and IL-12p70.

### Discriminatory model for sPTB

**Fig 4:**
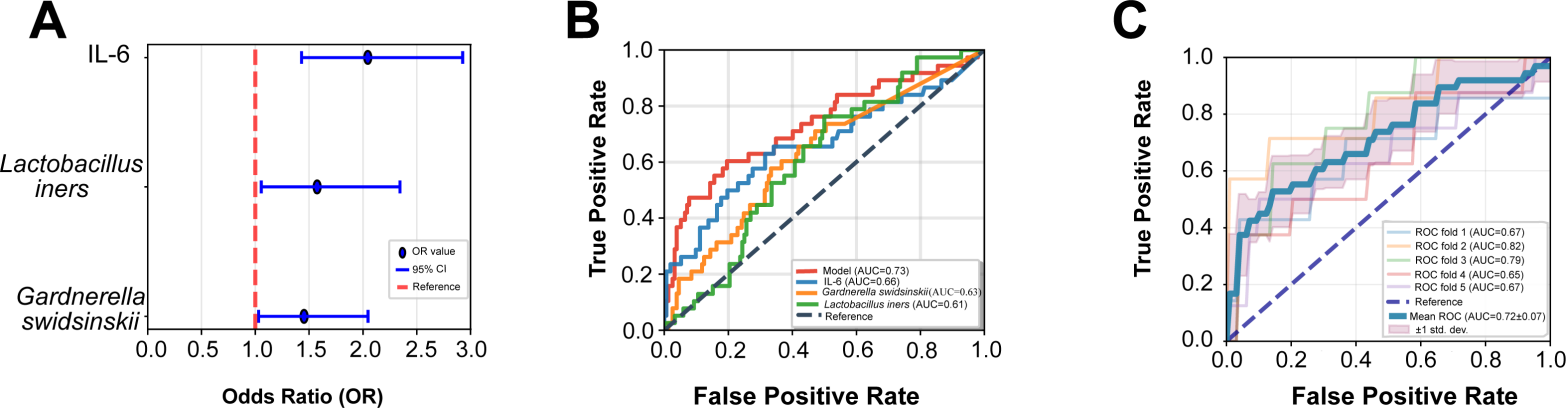
Logistic regression model for sPTB. (**A**) Adjusted odds ratios of predictors selected by LASSO regression: *Lactobacillus iners*, *Gardnerella swidsinskii*, and IL-6. (**B**) ROC curves for the logistic regression model combining IL-6, *G. swidsinskii*, and *L. iners*; and for each biomarker individually. (**C**) Fivefold cross-validation ROC curves for the logistic regression model. IL, interleukin; LASSO, least absolute shrinkage and selection operator; ROC, receiver operating characteristic; sPTB, spontaneous preterm birth.

Least absolute shrinkage and selection operator (LASSO) regression with L1 regularization analysis identified *L. iners*, *G. swidsinskii*, and IL-6 (all log-transformed) as significant risk factors for sPTB, with adjusted OR (95% CI) of 1.57 (1.06–2.34), 1.45 (1.03–2.05), and 2.05 (1.43–2.93), respectively (Fig. 4A). A multivariate logistic regression model for sPTB based on these three variables yielded an AUC of 0.73, which is higher than the AUC values for the individual variables: *L. iners* (AUC: 0.61), *G. swidsinskii* (AUC: 0.63), and IL-6 (AUC: 0.66) (Fig. 4B). Besides, the model’s performance was further evaluated by fivefold cross-validation, yielding a mean AUC of 0.72 ± 0.07 (Fig. 4C).

## DISCUSSION

This nested case-control study characterized the composition of the vaginal microbiome and cytokine profiles during mid-pregnancy in Chinese women and explored their association with sPTB. Generally, women experiencing sPTB exhibited increased abundance of vaginal *L. iners* and elevated levels of inflammatory cytokines in vaginal fluid. Furthermore, we established a discriminatory model combining bacterial taxa and cytokines, which demonstrated a fair discriminatory ability.

Evidences suggest high vaginal microbial diversity is associated with sPTB (4, 5). However, some studies did not find the association (14, 16, 20–22). A study in a predominantly African American cohort found no significant differences in richness or diversity between term and preterm deliveries at any trimester, although the vaginal microbiome was more stable over time in women delivering at term (16). Nelson et al*.* (14) even observed opposite findings: lower alpha diversity (phylogenetic diversity and Chao1) in nulliparous African American women with sPTB, although not significantly (*P* = 0.077 and 0.066). Similarly, in Chinese populations, Liu et al*.* (23) grouped PTB into preterm premature rupture of the membranes (PPROMs) and PTB without PROM, finding no significant difference in Chao1 index between PTB without PROM and term births, but PPROM individuals exhibited a lower Chao1 index than term births. Our study found no significant differences in alpha diversity index between sPTB and term groups. Discrepancies among studies may arise from variations in the ethnicity of population, timing of sampling, detection methods, and data processing.

The CST classification, which was first introduced by Ravel et al. in 2011 (24), is widely used to characterize vaginal microbial communities. Compared with *L. crispatus*, *L. gasseri*, or *L. jensenii*-dominated CSTs, which are recognized as optimal vagina status, *L. iners*-dominated CST (CST III) is more prone to vaginal dysbiosis (25), and related to sPTB (6). Although the distribution of CST showed no statistically significant difference overall between the term and sPTB groups in our study, the proportion of CST III was higher in the sPTB group compared to the term group (42.1% vs 28.9%). A proposed mechanism for *L. iners* linking to sPTB is that it produces d-lactic acid, which is less effective at inhibiting pathogens, while other *Lactobacillus* species mainly produce l-lactic acid to maintain the health of the vagina (25). Additionally, *L. iners* produce inerolysin, a pore-forming toxin related to vaginolysin of *G. vaginalis*, which can lyse host cells and release carbon sources (26). However, Wang et al. found that in the third trimester, the abundance of *L. iners* was higher in healthy pregnant women than in those with conditions, and *L. iners* strains isolated from healthy pregnant women can inhibit the growth of *G. vaginalis* (27). These highlight the critical importance of strain-level heterogeneity and the context-dependent nature of *L. iners*.

In previous studies conducted in China, one study found the relative abundance of *L. iners* was more abundant in pregnant women with PPROM (28); while two others found no difference in *L. iners* abundance between sPTB and control groups (29, 30). Our study demonstrated that high abundance of *L. iners* was associated with sPTB, and LASSO regression with L1 regularization identified high *L. iners* abundance as a significant risk factor for sPTB.

Intrauterine infection ascending from the vagina is an important hypothesized trigger of PTB (31). CST IV is not dominated by *Lactobacillus* spp., but comprises diverse bacteria often associated with BV (13) and has been linked to sPTB in Black, White, and Asian populations (10, 21, 32). However, we found no relationship between CST IV and sPTB. The discrepancy between our findings and previous reports (10, 21, 32) may be explained by heterogeneity in the populations studied, such as variations in ethnicity, geography, and risk factors. A recent study conducted in China found that although BV-associated taxa were related to high vaginal sialidase activity (which breaks down protective mucins), neither vaginal microbiome communities nor sialidase activities was associated with sPTB (29), highlighting differences in the vaginal microbiome and/or host immune response accounting for low sPTB rate in Chinese women.

Our study identified *G. swidsinskii* significantly related to sPTB. *Gardnerella* was originally known as *G. vaginalis*. However, high-resolution sequencing has led to the identification of 14 genomic species within the genus, with four formally named: *G. vaginalis*, *G. leopoldii*, *G. swindinskii*, and *G. piotii* (33). Munch et al. (34) found all four named *Gardnerella* species were more frequently detected in patients with BV. Another study targeting the V4 region of the 16S rRNA gene across seven cohorts observed that *Gardnerella* clades G1, G2, and G3 (which include *G. vaginalis*, *G. leopoldii*, *G. swindinskii*, and *G. piotii*) were associated with PTB (33). However, a recent Dutch study only found *G. leopoldii* significantly associated with PTB (35). The distribution of *Gardnerella* clades was influenced by both genomic and environmental factors (36). *G. swidsinskii* detection has been highly associated with recent vaginal intercourse (34). The correlation of *G. swidsinskii* abundance and sPTB should be interpreted cautiously due to the single-center nature and exclusive focus on Chinese women; further basic and clinical research is warranted.

Untimely activation of inflammatory response is linked to preterm labor, involving pro-inflammatory cytokines (37). IL-1β and IL-6 are significant cytokines in sPTB pathogenesis, playing roles in immune processes and amplifying inflammation by triggering other mediators (38). Cytokines have also been explored as biomarkers of sPTB (5, 10–12). Of the cytokines, elevated vaginal IL-6 level has consistently been associated with sPTB across different studies (5, 10–12), highlighting its values as a robust biomarker. Our findings agree with previous studies regarding cytokine roles, showing IL-1β, IL-6, and IL-12p70 were increased in the sPTB group relative to the term birth group. Furthermore, IL-6 was incorporated as a significant marker into the discriminatory model.

Our multivariate logistic regression model, based on *L. iners, G. swidsinskii,* and IL-6, yielded an AUC of 0.73, indicating fair discriminatory ability. This performance is comparable (39, 40) to or lower (11, 41, 42) than models in other studies. Discrepancies likely arise from multiple factors, such as ethics, individuals, and included parameters. sPTB is multifactorial, and infection is a principal factor, accounting for 25–40% of cases (3); the other features may also be involved. For instance, a model based on 20 differential genera across four cohorts (India, Japan, the United States, and Nigeria) achieved an AUC of 0.88 for predicting PTB, but validation across studies yielded AUCs ranging widely (0.52–0.98), and application to a Chinese cohort resulted in an AUC of only 0.638 (43), highlighting inter-study variability. Although achieving fair discriminatory ability, our model outperformed individual variables, demonstrating that combining bacterial taxa and cytokines enhances efficiency.

Compared to most previous studies using short-read 16S rRNA sequencing, we employed full-length 16S rRNA sequencing, which improves taxonomic sensitivity, specificity, and resolution for species-level identification (44, 45), such as distinguishing *Gardnerella* species. This technique facilitates more precise vaginal microbiome research.

However, there are some limitations in the present study. First, sample collection, which was only performed once from each participant during 16–28 weeks of GA, failed to reflect the dynamic changes of the vaginal microbiome during the pregnancy. Second, data on other potentially useful predictors (e.g., prior preterm birth, smoking history, and cervical length) were not available for analysis; their incorporation in future studies might improve discriminatory ability. Third, this single-center study has a relatively small sample size; multi-center studies with larger cohorts are needed to characterize the vaginal microbiome in Chinese women with sPTB.

### Conclusion

Overall, this nested case-control study revealed comparable alpha diversity and CST distributions in the vaginal microbiome of Chinese women delivering at term and those experiencing sPTB. However, women who experienced sPTB exhibited significantly higher relative abundances of *A. christensenii*, *G. swidsinskii*, and *L. iners*, along with elevated vaginal levels of IL-1β, IL-6, and IL-12p70. A model based on IL-6, *G. swidsinskii*, and *L. iners* demonstrated utility for sPTB discrimination. These findings suggest that specific alterations in the vaginal microbiome and inflammatory milieu may contribute to sPTB in the Chinese population.

## MATERIALS AND METHODS

### Study design

This was a nested case-control study performed at Women and Children’s Hospital of Ningbo University, Zhejiang province, China. Cases and controls were selected in a ratio of 1:4 from a prospective cohort, designed to investigate the association between vaginal microbiome and sPTB. Women were enrolled in the cohort if they had a singleton pregnancy between 16 and 28 weeks of pregnancy and were willing to participate in this study. Women were excluded if they met any of the following criteria: (i) women under 18 years of age, (ii) HIV positive status, (iii) multiple pregnancies, (iv) acute inflammation, and (v) severe systemic disease.

**Fig 5:**
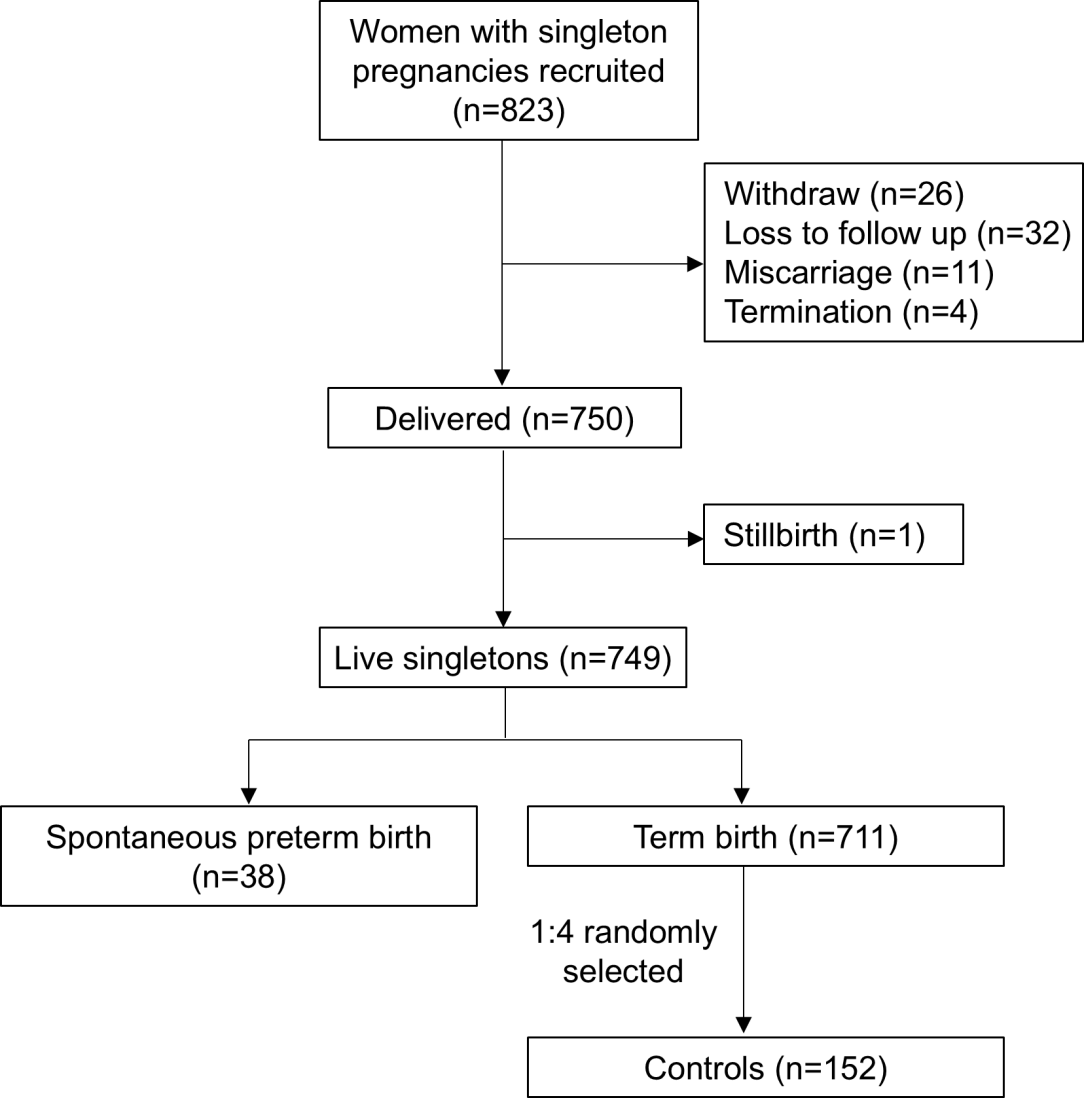
Flow diagram of selection of case and control from the cohort.

The initial cohort comprised 823 women with singleton pregnancies. After excluding 26 withdrawals, 32 losses to follow-up, and 16 pregnancies with miscarriage (*n* = 11), termination (*n* = 4), or stillbirth (*n* = 1), 749 women were eligible for study. Among them, 38 cases experienced sPTB. Consequently, 152 women with term delivery were selected randomly as controls (Fig. 5).

### Sample collection

At enrollment, two vaginal fluid samples were collected from the posterior vaginal fornix of each participant using a CLASSIQSwabs swab (COPAN Italia) without buffer. Samples were placed immediately on ice and frozen at −80°C within 5 min after collection until processing. Subjects were followed until delivery.

### DNA extraction, 16S rRNA gene amplification, and sequencing

Microbial DNA was extracted from vaginal swabs using the E.Z.N.A. Stool DNA Kit (Omega Bio-tek, Norcross, GA, USA). The V1-V9 regions of the 16S rRNA gene were amplified using primers 27F (5′-AGRGTTYGATYMTGGCTCAG-3′) and 1492R (5′-RGYTACCTTGTTACGACTT-3′), where the barcode is an eight-base sequence unique to each sample. Amplicons were purified using the AxyPrep DNA Gel Extraction Kit (Axygen Biosciences, Union City, CA, USA). SMRTbell libraries were prepared from the amplified DNA by blunt-ligation (Pacific Biosciences, Menlo Park, CA, USA). The purified amplicons were sequenced using the Sequel II Sequencing Kit 2.0 on a PacBio Sequel II platform (Pacific Biosciences).

### Processing of sequencing data

PacBio raw reads were processed using the SMRT Link Analysis software (version 11.0) to obtain demultiplexed circular consensus sequence (CCS) reads. Raw reads were processed through SMRT Portal to filter sequences for length (>1,000 or <1,800 bp) and quality. Sequences were further filtered by removing barcode and primer sequences with the lima pipeline (Pacific Biosciences demultiplexing barcoded software, https://lima.how/).

Passed sequences were dereplicated and subjected to the DADA2 algorithm (QIIME 2 recommended) to identify indel mutations and substitutions. The trimming and filtering were performed on paired reads with a maximum of two expected errors per read (maxEE = 2). Taxonomic assignment of each ASV was performed by aligning representative sequences against the NCBI Nucleotide Sequence Database using BLAST+ (Version 2.11.0) with the following criteria: *e*-value ≤ 1e⁻¹⁰, sequence identity ≥ 99%, and exclusion of non-standard/vague species annotations. This approach aligns with species-level classification protocols for full-length 16S rRNA data (46).

Alpha diversity indices, including Observed Species, Shannon index, and Simpson index, were calculated using Mothur v.1.21.1 after rarefaction to uniform depth (47). For beta diversity, a Bray-Curtis dissimilarity matrix was computed and visualized using PCoA.

CSTs were assigned to each sample on the basis of bacterial compositional similarity with Jensen-Shannon divergence and Ward, as previously described (24). CST I is dominated by *L. crispatus*, CST II by *L. gasseri*, CST III by *L. iners*, CST V by *L. jensenii,* and CST IV by a heterogeneous group lacking a dominant *Lactobacillus* species.

### Cytokine detection

Before tests, frozen vaginal swabs were thawed and suspended in phosphate-buffered saline (PBS). Cytokines, including interleukin (IL)-1β, IL-2, IL-4, IL-5, IL-6, IL-8, IL-10, IL-12p70, IL-17, interferon (IFN)-γ, IFN-α, tumor necrosis factor (TNF-α), were detected using flow cytometry (FACSCCanto II, Becton, Dickinson and Co., USA) with cytokine multiplex detection kit (CellGene Biotech Co., Ltd., China).

### Statistical analysis

For continuous variables, normally distributed continuous data were presented as means ± standard deviations (SDs), and non-normally distributed variables were expressed as medians and interquartile ranges (IQRs); for categorical variables, data were presented as percentages. The chi-square test, Fisher’s exact test, Mann-Whitney *U* test, and Student’s *t*-test were used for the comparison of demographics between sPTB and term groups. Fisher’s exact test was used to assess differences in CST distribution between groups. Microbial alpha diversity was measured using Observed species, Shannon, and Simpson index. Mann-Whitney *U* test was performed to assess the statistically significant difference in diversity indices between groups.

For bacterial taxa analysis, low-abundant taxa were filtered out. Taxa failing to meet the following criteria were removed: (i) relative abundance of ≥1% in at least 5% of samples, or (ii) relative abundance of ≥0.1% in at least 15% of samples (5, 10). Then, the value of relative abundance below 0.00001 were adjusted to 0. Differences in bacterial taxa abundance were determined using the Mann-Whitney *U* test.

For cytokines, those below the limit of detection in >30% samples were ruled out for analysis (5). Values below the limit of detection were imputed with the lower limit of detection for the eligible cytokines. The comparisons of cytokines were conducted with the Mann-Whitney *U* test. Associations between enrolled cytokines and bacterial taxa were assessed using Spearman’s correlation. FDR-adjusted *P* values < 0.05 were considered significant.

A multivariable logistic regression model for sPTB was developed. Eligible cytokines were log_10_-transformed (Table S2), and bacterial taxa relative abundance were log-transformed as log_10_ ([relative abundance + 0.001]/0.001) (Table S3). Variable selection for the model proceeded in two stages. First, among variables (demographics: gravidity, parity, previous miscarriage, age > 35 years; CSTs, eligible cytokines and bacterial taxa), we performed univariate screening (*P* < 0.1) to identify candidate variables (Table S4). Second, we applied LASSO regularization with L1 penalty to select the most predictive features. Standard logistic regression was then fitted using these selected features. The discriminatory ability of this model was evaluated by the area under the receiver operating characteristic curve (AUC), and validated using fivefold cross-validation.

Statistical analyses were performed using the Python scikit-learn package and SPSS statistical 26.0 software (IBM, Armonk, NY, USA). *P* <0.05 was considered statistically significant.

## Data Availability

The sequencing data of this study can be found at the Sequence Read Archive (accession code PRJNA1080493). The other data generated or analyzed during this study are included in this article and its supplemental material. The sample name in the database is consistent with number in Table S1.

## SUPPLEMENTAL MATERIAL

Supplemental material spectrum.02607-25-s0001.xlsx Tables S1 to S9.

ASM does not own the copyrights to Supplemental Material that may be linked to, or accessed through, an article. The authors have granted ASM a non-exclusive, world-wide license to publish the Supplemental Material files. Please contact the corresponding author directly for reuse.
